# Wave Propagation in Aluminum Honeycomb Plate and Debonding Detection Using Scanning Laser Vibrometer

**DOI:** 10.3390/s18061669

**Published:** 2018-05-23

**Authors:** Jingjing Zhao, Fucai Li, Xiao Cao, Hongguang Li

**Affiliations:** State Key Laboratory of Mechanical System and Vibration, Shanghai Jiao Tong University, Shanghai 200240, China; zjj316@sjtu.edu.cn (J.Z.); caoxiaojx@sjtu.edu.cn (X.C.); hgli@sjtu.edu.cn (H.L.)

**Keywords:** guided wave, honeycomb sandwich structure, debonding detection, scanning laser vibrometer

## Abstract

Both the aerospace and marine industry have widely relied on a honeycomb sandwich structure (HSS) because of its high strength-to-weight ratio. However, the intrinsic nature of an adhesively bonded multi-layer structure increases the risk of debonding when the structure is under strain or exposed to varying temperatures. Such defects are normally concealed under the surface but can significantly compromise the strength and stiffness of a structure. In this paper, the guided wave method is used to detect debondings which are located between the skin and the honeycomb in sandwich plates. The propagation of guided waves in honeycomb plates is investigated via numerical techniques, with emphasis placed on demonstrating the behavior of structure-based wave interactions (SWIs). The SWI technique is effective to distinguish heterogeneous structures from homogeneous structures. The excitation frequency is necessary to generate obvious SWIs in HSSs; accordingly, a novel strategy is proposed to select the optimal excitation frequencies. A series of experiments are conducted, the results of which show that the presented procedure can be used to effectively detect the locations and the sizes of single- and multi-damage zones in HSSs.

## 1. Introduction

A sandwich structure consists of two strong panels (referred to as skin) and a lightweight core, which are joined together by adhesive bonding films. In comparison with isotropic aluminum materials, a notable advantage of aluminum sandwich plates is their high strength-to-weight ratio, which provides an efficient mechanism for resisting bending and buckling loads [1,2]. Because of its lightweight yet strong property, the honeycomb sandwich structure (HSS) is one of the most common sandwich structures used in marine, aerospace, and aeronautic industries [3,4]. Nevertheless, as a result of its inherent weakness in terms of adhesive bonding, both intensive and cyclic loads on HSSs can introduce debondings along the skin-core interfaces, substantially compromising its strength and stiffness [5]. Since debonding is concealed under panels, it is difficult to detect. Therefore, it is necessary to develop an effective monitoring system for debonding detection in HSSs.

Guided waves (GWs) have a high sensitivity to both surface and internal structural damages and have been extensively used to develop various damage identification algorithms in the evaluation of small damages in both composite and metallic materials. Su etc. [6] provided a comprehensive review on the state of guided wave-based damage identification approaches for composite structures, addressing both the field’s advances and achievements. Additional research was conducted to verify the potential capability of GWs to monitor complex structures [7,8,9,10,11,12,13]. Over the past two decades, GWs have been used for nondestructive materials testing [14], but the application of the waves propagation in heterogeneous plates, such as in HSSs, remains in a preliminary stage. It is often very difficult to predict the propagation of GWs in HSSs through theoretical approaches as a result of complex heterogenic geometry [15]. Thus, most studies have focused on utilizing numerical simulation (such as finite element analysis, FEA) to investigate the propagation characteristics of GW and the damage detection method in HSSs. 

Identification of the invisible debondings at the skin-core interface is particularly difficult in HSSs. Current techniques for identifying the debonding in HSSs can be classified into three main categories: one, changes of time-of-flight (ToF), which are induced by the boundary reflection of debonding on the basis of the group velocity; two, the phenomena of leaky GWs, demonstrated by the energy attenuation of waves propagation; three, changes to the signal of GWs, acquired by transducers through signals processing. For example, Diamanti etc. [16] developed a cost- and time-effective strategy for inspecting the impact damage in monolithic beams as well as the debonding in the sandwich beams using the ToF of low-frequency GWs. Mustapha et al. [17] assessed debonding locations using the damage-reflected ToF and evaluated the severity of debonding using both the magnitude of the reflecting wave signal and the delay in the ToF of GW signals. However, the ToF technique relies heavily on group velocity dispersion curves. The sensitivity and revolution of the ToF technique is limited by both the size and location of damages. Firstly, the reflected wave from the damage cannot be identified when the damage is close to the boundary [16]. Secondly, the amplitude of the reflected wave from the damages is not clear when the edges of honeycomb cell in HSSs cause unclear, scattered waves [18] or the wave signals are attracted by electrical noise with a low signal-to-noise ratio [15]. The disturbance and distraction become more conspicuous when the honeycomb sandwich plates are made of metal. The scattered waves contain more energy and have greater amplitudes due to the high acoustic impedances of the metal cores [18], which lowers the amplitude of reflected waves.

The phenomenon of leaky GWs is a natural effect of the propagation of GWs in sandwich plates, which was first observed in 1982 [11]. When GWs propagate in HSSs, the energy attenuates quickly because of the loss of energy leaking into the cores. When the GWs propagate in bonded sandwich plates, the cores leak energy, making the amplitudes of reflected wave lower; in contrast, in the debonding area, the amplitudes remain relatively greater. Qi et al. [19] compared GW transmission energy between the undamaged and the damaged models to identify skin-core debonding. As the results show, the fundamental anti-symmetric mode ($A_{0}$ mode) of Lamb waves is more sensitive to the debonding than the fundamental symmetric mode ($S_{0}$ mode) [15] when the debonding detection techniques depend on the differences to the change in energy or amplitude between the bonded zones and debonded zones. This is attributable to dissipation of more energy in $A_{0}$ mode into the cores of the bonded HSSs when compared to $S_{0}$ mode. Mustapha [20] investigated the leaky and non-leaky behaviors of GWs, focusing on the fundamental Lamb wave mode and Rayleigh waves. The characteristics of leaky and non-leaky GWs as they interacted with debonded zones were explored. Results indicated that Leaky waves were effective for debonding detection in composite materials. Generally, several factors impact the effectiveness of leaky waves in the detection of debondings in HSSs. Firstly, the appropriate and sensitive Lamb wave mode and frequency must be selected to detect the exact debonding zones in HSSs, which is important for detecting the skin-core debonding confirmed in the studies mentioned above [21]. Secondly, the differences of acoustic impedance between the skins and cores as well as the skin-to-core thickness ratio significantly affect features of leaky waves, which are expressed by the wave attenuation curve [15,20]. Moreover, the wave attenuation curve of either the specimen or the benchmark must be a transcendental condition, when leaky waves are used to inspect damages in HSSs.

The Reconstruction Algorithm for the Probabilistic Inspection of Defects (RAPID) is a tomography technique for damage detection. The technique is based on the signal difference coefficient (SDC); however, it does not extract damage features from raw signals, which is a component of the previously mentioned techniques. Song and Mustapha [15,22] successfully applied the RAPID on debonding detection in HSSs. The results provide reliable quantitative information about the location and size of the debonding in HSSs without extracting the damage feature. However, RAPID has two predominant disadvantages. The first is that the undamaged state must be taken as a reference; the second is that additional effort is needed to eliminate the pseudo-damage image which results from the sensor network strategy. Additionally, the experiment environment must be stable; even a minimal change in temperature can induce errors [18]. Therefore, it is necessary to develop a novel method that can be carried out without benchmark and reference states and which also does not suffer from the drawbacks of currently available techniques. 

Recently, efforts have been made to acquire the propagation properties of GWs for monitoring debondings in HSSs. The dispersion curves of the homogeneous plates can be accurately obtained from Rayleigh-Lamb equations [23], while for heterogeneous plates accurate dispersion curves can only be obtained by numerical calculations. The semi-analytical finite element method (SAFEM) combines the advantages of analytical method and finite element method, which is applied on HSSs as has been demonstrated by several studies [7,24,25]. Hence, finite element models, with the detail of geometry developed by commercial finite element software, e.g., LS-DYNA, ABAQUS, etc., have been used to solve the GW propagation problem in HSSs [15]. As a result of the complex structures of HSSs, the geometry of HSSs [26] is commonly simplified or equivalent parameters are used [27], rather than actual structural parameters, to reduce computing costs. However, in so doing, the dynamic of honeycomb cells in HSSs is ignored. However, the agreement between the GW propagation properties in the actual structure model and the simplified model depends on the excitation frequency [15,27]. The limitations of simplification become clear when the wavelength is almost the same as the characteristic length scale of the cellular microstructure of HSSs. This can be explained the interaction of the waves with the cellular microstructures [26]. The characteristics of interactions between the waves and the cellular microstructures, which are the unique properties of GW propagation in the HSSs, have yet to be studied in detail. GWs exhibit unique features when they are propagating in the HSSs at an appropriate range of frequency [15,27]. The wave fronts of GWs propagation have the same phase, with the same amplitude, in homogeneous materials. However, as Tian’s study has demonstrated, the amplitude is different in the same wave front in HSSs, which is a result of structure-based wave interactions (SWIs). The results of both the simulation and the experimental test showed the existence of complex SWIs where the waves interacted with the honeycomb cores [28]. Both the reason for the occurrence of SWIs and the factors which impact the characteristics of SWIs requires additional research.

In this paper, the properties of SWIs are studied sufficiently and the relationship between the geometry of HSSs and the excitation frequency is established. Then a debonding detection technique without reference samples is developed based on how SWIs is caused to distinguish debonding zones from the perfectly bonded zones, without concerning about group velocity and priori conditions. In our technique, it proves that the excitation frequency plays a vital role in the debonding detection technique. A three-dimensional finite element model is created using the commercial finite element software ABAQUS. The experimental studies are conducted using the proposed technique to verify the numerical simulation and then assess locations and sizes of the debondings in HSSs. A scanning laser vibrometer (SLV) acquires the sufficient information of wave fields in the experiments.

## 2. The Selection of Excitation Frequency

On the basis of current literature, a brief introduction to understand how the GWs propagate in HSSs is offered [15,28]. When the frequency is relatively low, GWs are generated and propagate in the whole composite structure. GWs subsequently propagate in the skins at incremental frequencies. The waves which propagate at a relatively high frequency can be regarded as leaky GWs, propagating in the skins of HSSs [21]. The wave fronts of GWs generally form concentric circles with the same amplitude when the waves propagate in the HSSs at a high frequency. At a particular frequency, complex SWIs are induced via the interaction of the waves with the cellular microstructures of honeycomb, which do not exist in homogenous material structures. Accordingly, even at the same frequency, the SWIs do not exist when GWs propagate in debonding areas of HSSs because a debonding area can be regarded as homogenous material. In this section, we will discuss the specific frequency range for the occurrence of SWIs and establish the relationship between the excitation frequency and the geometric parameters of honeycomb plates.

To research the frequency range in which the SWIs appear in HSSs, the numerical simulation technique and finite element method is used. In this study, the GWs in HSSs were simulated by the commercial finite element software, Abaqus/CAE 6.13-1. Through the finite element method, the GWs at different frequencies were simulated and compared.

**Figure 1:**
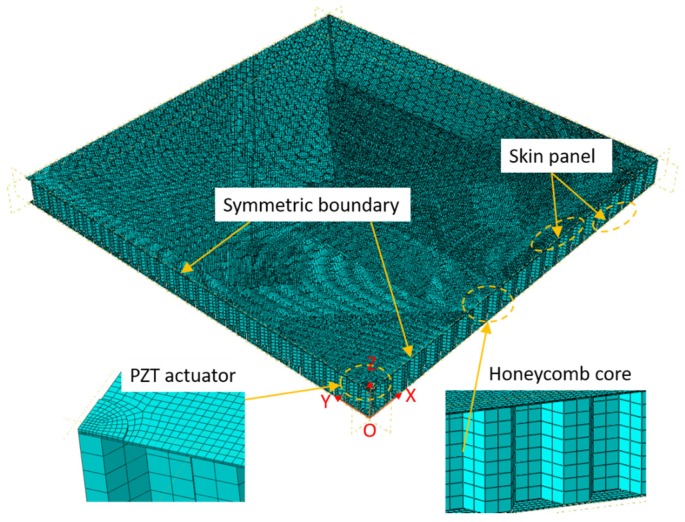
The finite element model of a HSS with a surface-bonded PZT.

Figure 1 shows the finite element simulation setup from a honeycomb sandwich plate with a surface-bonded piezoelectric wafer (PZT). The honeycomb sandwich was composed of two aluminum skin plates and an aluminum honeycomb core. A round PZT actuator was bonded on the top surface of the structure to generate GWs. The geometric parameters of the model are listed in Table 1. The skin plates and honeycomb cores had the same material properties with Young’s modulus 70 GPa, Poisson’s ratio 0.33, and density 2700 kg/m^3^.

The finite element model was built in the session of standard/explicit model in the commercial software Abaqus/CAE 6.13-1. To simulate the GWs propagation in the HSS, transient analysis was used. However, the piezoelectric element could not be used in the transient analysis. Accordingly, a thin aluminum cylinder was used instead of the PZT to provide uniform force along the cylindrical wall. For a symmetrical model, only a quarter of the honeycomb sandwich was built, as shown in Figure 1. Symmetric boundary conditions were applied on all nodes on the two symmetric planes (O–X–Z plane and O–Y–Z plane). In the finite element model, S3D8R elements were used to build the skin plates. S4R elements were used to build the honeycomb core with hexagonal cells. The load excitation was five-count tone-burst signals enclosed in a Hann window with different center frequencies of 15 kHz, 35 kHz, 45 kHz, and 80 kHz. In the transient analysis, a small element size was enforced on the finite element model to accurately capture the GWs propagating in the HSS. The element size needed to be small enough to guarantee that there were at least ten elements per wavelength along the wave propagation direction [21,29]. Moreover, the integration time step needed to be small enough to ensure stability of the transient analysis [15].

**Figure 2:**
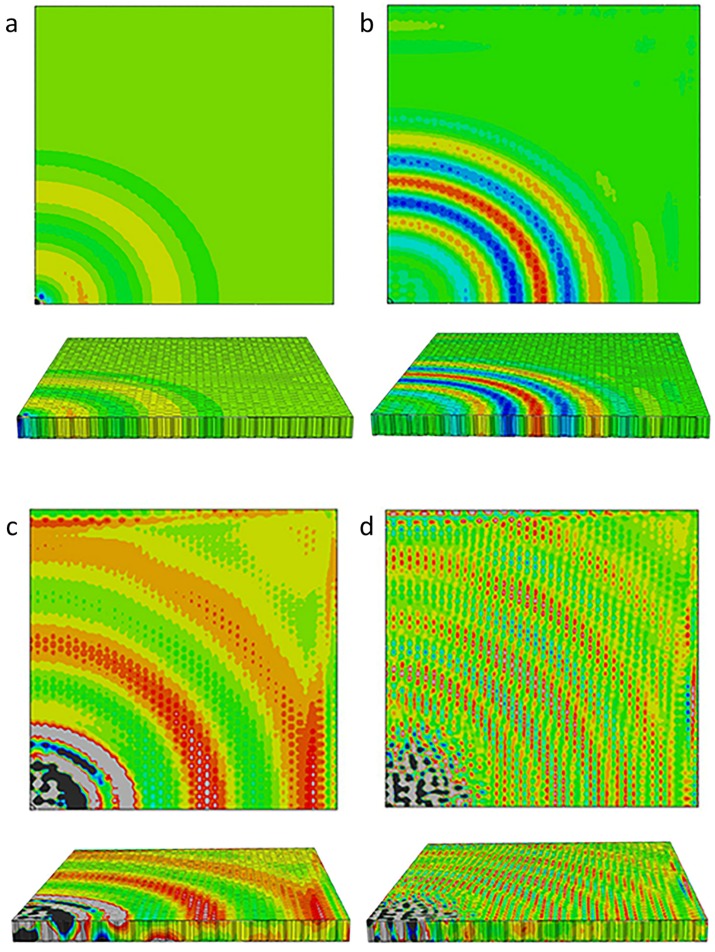
Simulated GW-field of displacement in Z direction: (**a**) top and front view for 15 kHz excitation at 230 μs; (**b**) top and front view for 35 kHz excitation at 230 μs; (**c**) top and front view for 45 kHz excitation at 120 μs; (**d**) top and front view for 80 kHz excitation at 120 μs.

The GWs in the honeycomb sandwich at four different frequencies, 15 kHz, 35 kHz, 45 kHz, and 80 kHz, were simulated. Figure 2 provides the simulation results of wave fields in Z direction. With frequencies of 15 kHz and 35 kHz, the simulation results in Figure 2a,b demonstrate the global GWs propagating in the entire sandwich plate based on a frontal view. In both instances, the entire plate can be considered as a waveguide that supported the global GWs. At 45 kHz, the global waves disappeared in which the waves propagated only in the skins instead of the global GWs. Similar to GW propagation in homogeneous structures, the GWs were composed of two modes, quasi-S0 and quasi-A0 [16,17,21]. At the highest frequency, 80 kHz, waves interacted with the microstructures of the honeycomb cellular, activating the SWI behaviors (as shown in the detail of the wave field). A comparison of the results of the simulation in different frequencies indicated that the complex SWIs indistinctly appeared at 35 kHz, with the SWIs becoming more distinct as frequency increased. On the basis of this phenomenon, the occurrence of SWIs and the excitation frequency are clearly closely related.

**Figure 3:**
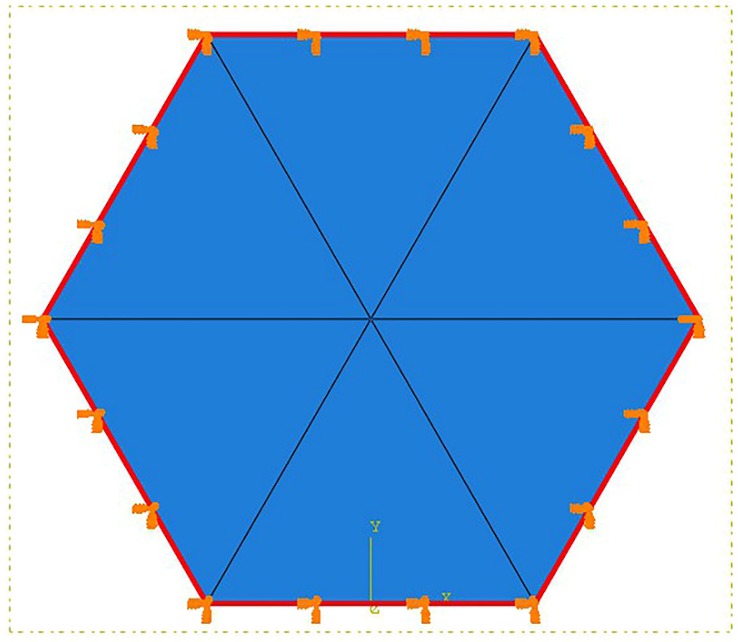
The finite element model of the hexagon surface of honeycomb.

**Figure 4:**
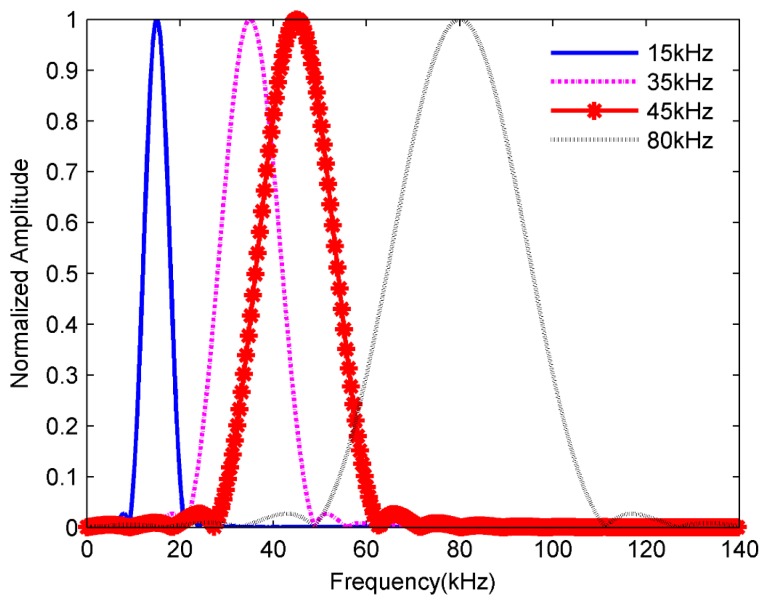
The excitation signals in the frequency domain.

The numerical model of a hexagon surface corresponding the honeycomb structure was built to calculate its natural frequencies and mode shapes. The finite element model, interlaced with C3D15 elements, is shown in Figure 3. In consideration of the geometry of the HSS, the boundary of the hexagon surface was fixed. The first ten order natural frequencies of hexagon surfaces calculated from the element model are presented in Table 2. The excitation signals with different frequencies are presented in the frequency domain in Figure 4 to determine the relationship with the natural frequencies of the hexagon. From the simulation results in Table 2, 15 kHz is below the first-order mode frequency and the SWIs do not appear. When the excitation frequency was 35 kHz, the SWIs appeared (see Figure 2b). In this case, the first-order natural frequency was within the excitation frequency band because of the form of narrow-band excitation signal. As shown in Figure 2c, the SWIs became distinct at a frequency of 45 kHz, which is higher than the first-order natural frequency, 37.59 kHz, which is contained in the excitation frequency band. When the excitation frequency rises to 80 kHz, the SWIs continued to exist and consisted of discontinued wave fronts. These results show that the complex SWIs appeared when the excitation frequency band contained the first-order natural frequency. Tian et al. [28] also verified that the SWIs appeared at a frequency between 50 kHz and 100 kHz. On the basis of the geometry of HSSs in that study, the honeycomb cell size was 6.35 mm, and the thickness of skins was 1 mm. The first-order natural frequency of the honeycomb hexagon surface was 72.48 kHz, which was calculated using the numerical model with fixed boundary conditions. The relationship between the complex SWIs and the natural frequency of the honeycomb cell was verified both in the simulation and experiments in Tian et al. [28].

**Figure 5:**
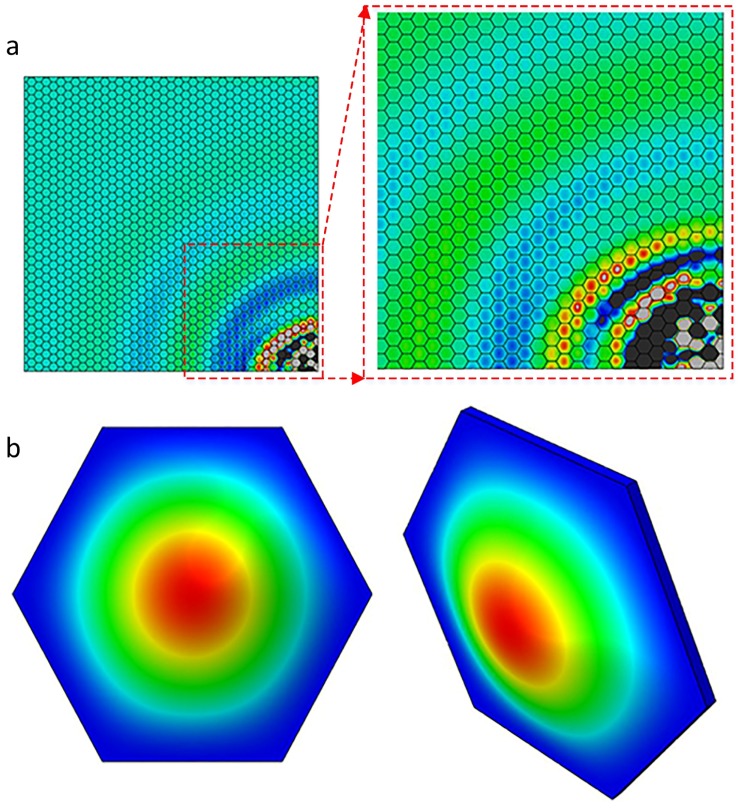
The relationship between the SWIs and the honeycomb core displacement in Z direction: (**a**) back view of the top skin at 45 kHz; (**b**) the first-order mode shape of the hexagon surface.

**Figure 6:**
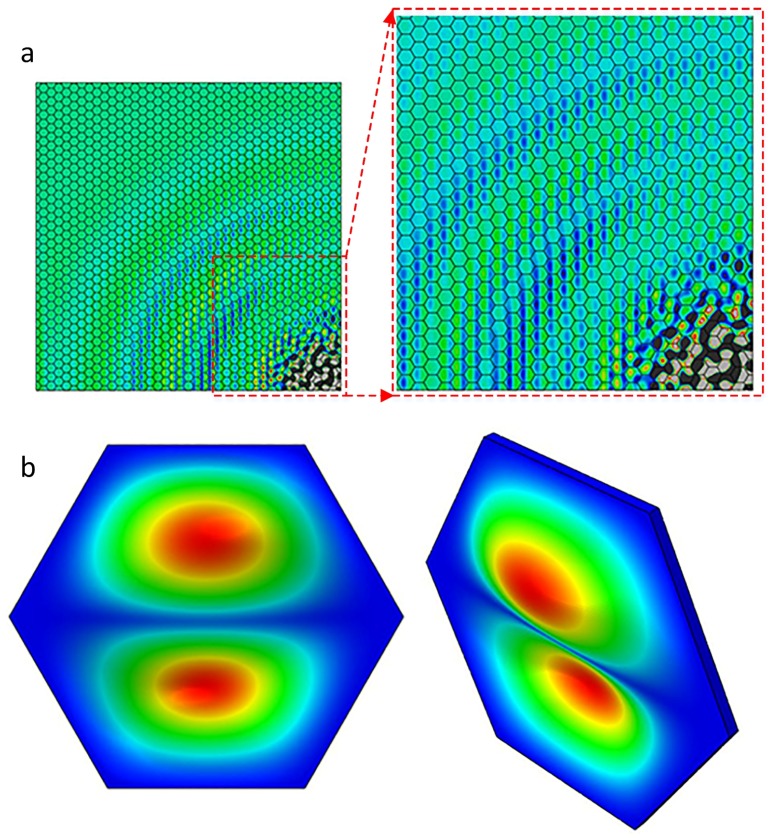
The relationship between the SWIs and the honeycomb core displacement in Z direction: (**a**) back view of the top skin at 80 kHz; (**b**) the second-order mode shape of the hexagon surface.

Additionally, SWIs formed a regular pattern which corresponds to geometric property of the cellular microstructures in honeycomb plates. Figure 5 and Figure 6 show the back view of the top skins in HSSs at frequencies of 45 kHz and 80 kHz, respectively. In Figure 5, the amplitude of the vibration reached its peak in the middle of a hexagon surface, which corresponded to the first-order mode shape of the hexagon with a single peak. The similar phenomenon occurred in the second-order mode shape of the hexagon, as shown in Figure 6. The second-order mode shape of the hexagon had two peaks: one was a crest of waves and the other was a trough of waves. The two peaks of Z direction appeared on the honeycomb cell surface simultaneously. When the excitation frequency band contained the first-order natural frequency, resonance occurred. The amplitude of the vibration was dramatically enlarged in the middle of hexagonal surface that was bonded with honeycomb core. The amplitude of hexagonal edges was restricted by its bond with honeycomb. The periodic change of amplitude depended on the regular honeycomb structures which formed regular patterns and was according to the nature of SWIs. Therefore, the wave field of SWIs in Z direction corresponded to the geometric properties of the cellular microstructures and the mode shapes of the honeycomb hexagon.

Notably, another important feature revealed the relationship between the excitation frequency and the behavior of mode shape in a cell. The first-order mode shape appeared when the excitation frequency was 35 kHz and 45 kHz, while the second-order mode shape appeared when the excitation frequency was 80 kHz. On the basis of the natural frequency shown in Table 2, 45 kHz was between the first- and second-order natural frequency, while 80 kHz was higher than the second-order natural frequency. This suggests that the mode shape was excited by the excitation frequency that was closest to the natural frequency.

In conclusion, the phenomenon of the SWIs adheres to the following rules: one, the first natural frequency of the honeycomb hexagon is a component of the excitation frequency in frequency domain; two, mode shapes are prone to be excited by the frequency component that is closest to the natural frequencies; three, the Z direction wave field of a single honeycomb hexagon corresponds to its mode shape. Notably, the Z direction wave-fields did not present strictly regular patterns when the excitation frequency was relatively high, presumably because the higher mode shape exhibited additional peaks which led to more complex wave fields in each hexagon.

It is necessary to select an appropriate excitation frequency to detect debondings in the HSSs. The SWIs, regarded as the predominant feature of HSSs in distinguishing the perfect bonding zones from the debonded zones, offer a novel tool to detect damage in HSSs. On the basis of the discussion in this section, the SWIs comprised regular patterns relating to the mode shapes excited by the excitation frequency. The lower order mode shapes exhibited fewer peaks to make the patterns obvious, while the higher order mode shapes displayed more peaks to make more complicated patterns. The complicated patterns disturbed the reflected waves from the damage edges, increasing the difficulty in identifying the damaged areas.

## 3. Experiments in Debonding Detection in HSSs

The GWs in a honeycomb sandwich plate ere measured by means of laser vibrometer tests. The experimental measurements were used to verify the feature of SWIs in a finite element simulation. Moreover, the experimental measurements of SWIs detected multi-damage zones in honeycomb sandwich plates.

### 3.1. SLV Tests Setup

Laser-based GWs which sense damage detection in composite structures are not innovative tools [30,31] but still present numerous challenges [32]. Defects and damages are usually identified by a change of the responding signal in terms of signal attenuation, arrival time, and/or mode conversions [33]. The anisotropy of composite structures makes any interpretation of the signals, which determine defects and damages, difficult. One of the main advantages of laser-based sensing is the high control speed [34]. Currently, both one-dimensional and three-dimensional SLVs are used for impact damage detection in composite structures. However, even if 3D lasers offer additional potential in terms of in-plane and out-of-plane wave-field components, one-dimensional lasers are more frequently chosen for debonding detection in HSSs. A one-dimensional laser vibrometer is easier to use through simple alignment and focusing; it is also more cost effective in terms of industrial applications. Additionally, the proposed strategy of excitation frequency selection in this study allows damage detection to use only the out-of-plane wave-field components in HSSs.

**Figure 7:**
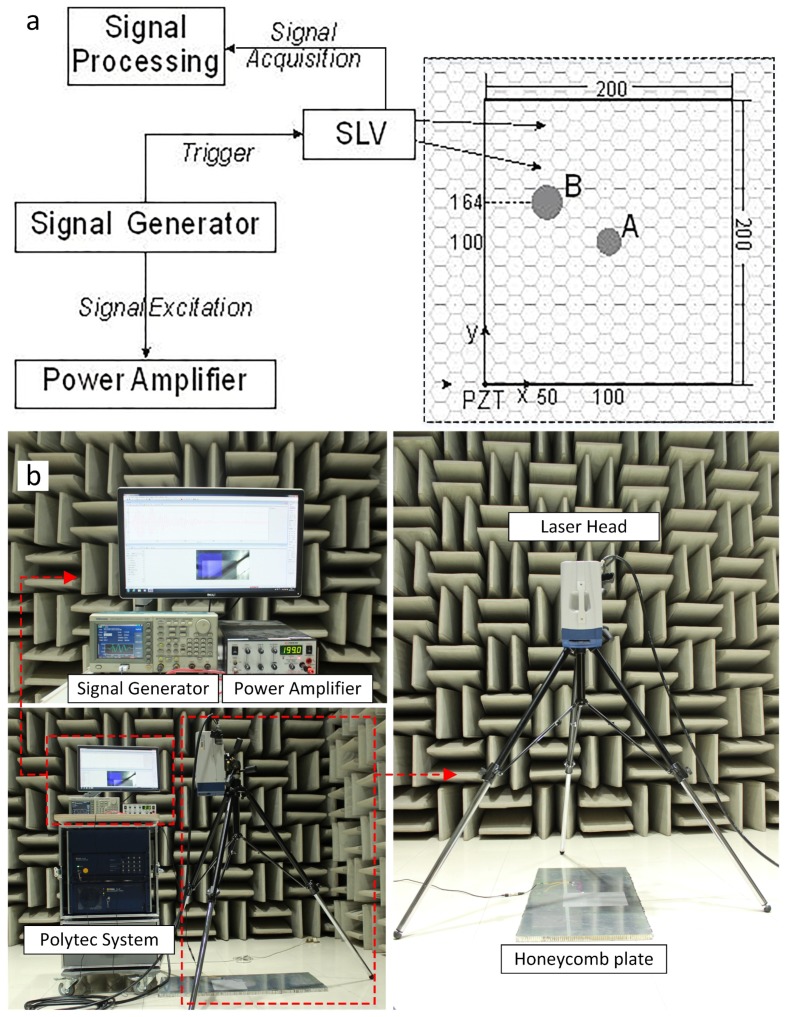
The SLV tests: (**a**) the schematic diagram of tests. (**b**) the experimental figure.

SLV tests were performed to acquire the GW fields and visualize the GW propagation. A one-dimensional SLV (Polytec PSV-500) was used to acquire the time-space wave field over the scan area (shown in Figure 7). In this test, the SLV was positioned normal to the plate surface such that only the out-of-plane wave velocities were measured and analyzed. Scanning was performed in the 200 mm × 200 mm scan area, with a spatial resolution of 2.5 mm in rough. At each point, the measurement was averaged 30 times to improve the signal-to-noise ratio (SNR). To further enhance the SNR, a retro-reflex foil was applied to the scan area. Previous studies have shown that the usage of foil does not influence the measured displacement, when the frequency is kept below 500 kHz [35].

### 3.2. Sample Preparation

Two honeycomb sandwich planes were manufactured for the tests, with top and bottom aluminum alloy skins (T6001 aluminum alloy with 0.3 mm) and the hexagonal-called 0.03 mm aluminum core. The geometry parameters of the honeycomb core were the same as the honeycomb core parameters listed in Table 1. The samples were cut into 900 mm × 500 mm sections, with a square scan area, as shown in Figure 7. One PZT (P.I^®^PIC255) of 10 mm diameter and 1 mm thickness was surface-mounted onto each sample, as an origin of the coordinate system. The PZT served as the actuator to generate GWs. One of the samples (D1) had no debondings. The other (D2) had two debondings (debonding A and debonding B). Debonding A was a 20 mm diameter circle at (100,100). Debonding B was a 26 mm diameter circle at (50,164). The summary of the details about debondings is shown in Table 3. The debondings were introduced during fabrication by removing the honeycomb cores.

### 3.3. Experimental Setup

Generation and acquisition of GWs consisted predominantly of an arbitrary signal generator (Tektronix^®^TEK AFG3022B), a signal power amplifier (Krohn-Hite^®^ KH 7602M), and an oscilloscope (Tektronix^®^DPO 3014). Five-cycle sinusoidal tone bursts, enclosed in a Hann window, were generated and applied to the PZT actuator; the GW signals were captured using the SLV at a sampling rate of 256 kHz. On the basis of the discussion in the previous section, 15 kHz, 45 kHz and 80 kHz were all used as excited frequencies in the experiments. All specimens lay on the floor without any clamping. Any clamping of the specimens would cause a change in their stiffness, making it difficult to reproduce the experimental results unless the exact amount of applied torque could be controlled.

## 4. Results and Discussion

### 4.1. The Relationship between Excitation Frequencies and the SWIs

**Figure 8:**
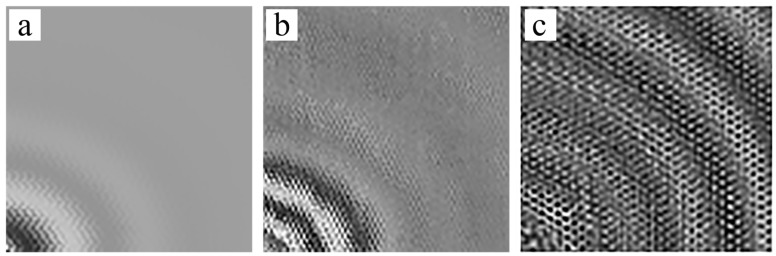
The wave field measured by SLV at 80 μs: (**a**) 15 kHz; (**b**) 45 kHz; (**c**) 80 kHz.

To verify the feature of SWIs in the honeycomb plates, excitation frequencies of 15 kHz, 45 kHz, and 80 kHz were selected in D1, respectively. 15 kHz was below the first-order natural frequency, and the excitation frequency band did not include the first-order natural frequency. 45 kHz was slightly higher than the first-order natural frequency, and the first-order natural frequency was one of the components in frequency domain. Figure 8 shows the results of the GW propagating in the scan area that was measured by the SLV. The GWs were excited by the PZT at the left bottom corner.

Figure 8a presents the GW field at 80 μs. No SWIs appeared when the excitation frequency was 15 kHz. SWIs appeared when the excitation frequency was 45 kHz, as shown in Figure 8b. The SWIs were more evident at 80 kHz, as shown in Figure 8c. Figure 8a–c correspond to the results of simulation in Figure 2a,c,d, respectively, but were limited by the spatial resolution of SLV (around 2.5 mm). Therefore, the second-order mode shape of the hexagonal surface is indistinct in Figure 8c and the honeycomb structure is only vaguely seen in Figure 8b. The experiment was designed to verify whether SWIs occur when the excitation frequency was close to or higher than the first-order natural frequency. The out-of-plane wave field in a single honeycomb hexagon related to the first-order mode shape based on the details shown in Figure 8b.

### 4.2. The Multi-Damage Zones Detection Test

Sample D2 exhibited two damages in HSS when tested with the excitation frequencies of 15 kHz, 45 kHz, and 80 kHz. The three frequencies represented three cases: the excitation frequency below the first-order natural frequency, that close to the first-order natural frequency, and that near the second-order natural frequency of the hexagon. No SWIs appeared at 15 kHz. With 45 kHz excitation, the SWIs exhibited a typical first-order mode shape of the hexagonal surface. The second-order mode shape as excited at 80 kHz with two peaks in one hexagon, making the patterns more complicated.

**Figure 9:**
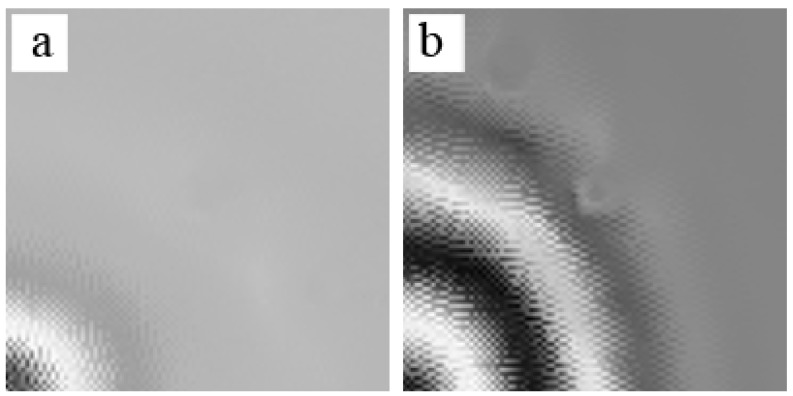

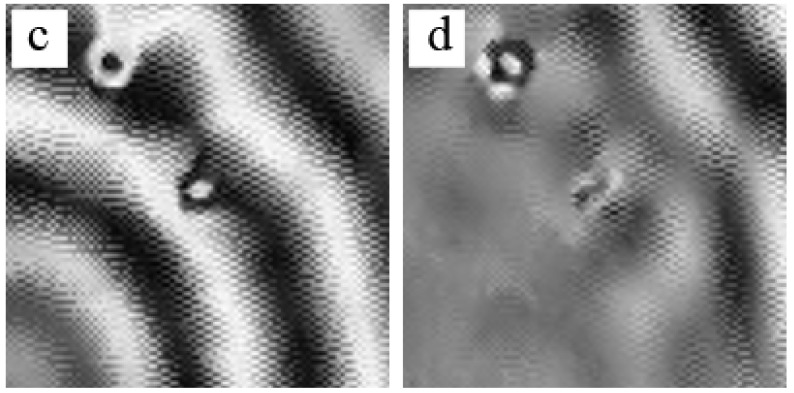
Experimental results at the frequency 15 kHz for D2, with two debondings at the locations of (100,100) and (50,164): (**a**) wave field at 80 μs; (**b**) wave field at 240 μs; (**c**) wave field at 400 μs; (**d**) wave field at 560 μs.

Figure 9 demonstrates the GWs propagating in D2 as well as the results of multi-damage zones detection at the excitation frequency of 15 kHz. At 80 μs, the GWs were generated from the PZT actuator in Figure 9a. At 240 μs, the incident waves arrived at debonding A while the wave fronts were interrupted by the debonding (Figure 9b). The GWs generated by PZT consisted of two components. One as quasi-$A_{0},$ with strong incident wave fronts and short wavelengths. The other component was quasi-$S_{0},\;$with weak wave fronts and long wavelengths. Because the SLV as positioned in front of the measurement surface, a predominantly out-of-plane vibration component was obtained. Unfortunately, the quasi-$S_{0}$ mode had only a small out-of-plane component and as imaged weakly, whereas the quasi-$A_{0}$ mode exhibited a strong out-of-plane component and was clearly imaged. In this case, the group velocities of waves propagating in the HSSs at relatively low frequencies approximated skin-based propagations [21,27]. On the basis of the Rayleigh-Lamb dispersion equations, the analytical values of the group velocity at 15 kHz was 5398 m/s for $S_{0}$ mode and 417 m/s for $A_{0}$ mode. The distance for wave propagation during 240 μs was 1296 mm for $S_{0}$ mode and 100 mm for $A_{0}$ mode. Taking the impact of the narrow-band signal and dispersion into consideration, the GWs in honeycomb plates measured by SLV contained only a quasi-$A_{0}$ mode component because its energy was predominantly concentrated on the out-of-plane vibration. The GWs covered the entire scanned area at 400 μs, as shown in Figure 9c. Additionally, the reflected waves were produced by interactions with the edges of the debondings. The damage zones obviously distinguished the debondings from the perfect zones. The reflected waves vanished at the same time as those which disappeared in Figure 9d. Because of the processing of the propagation of GWs in the honeycomb plates, no SWIs appeared.

**Figure 10:**
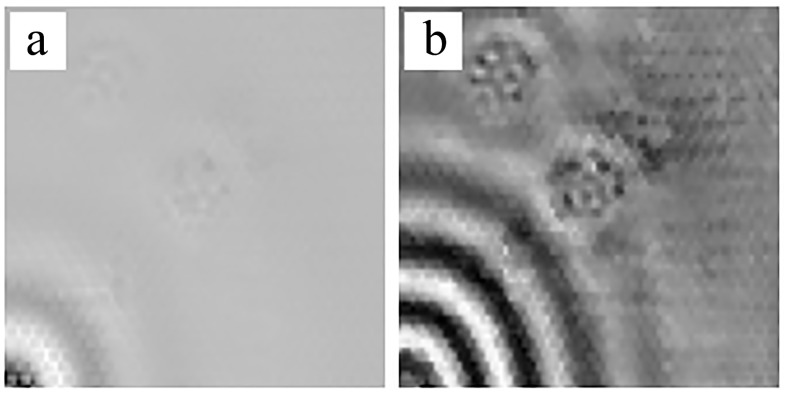

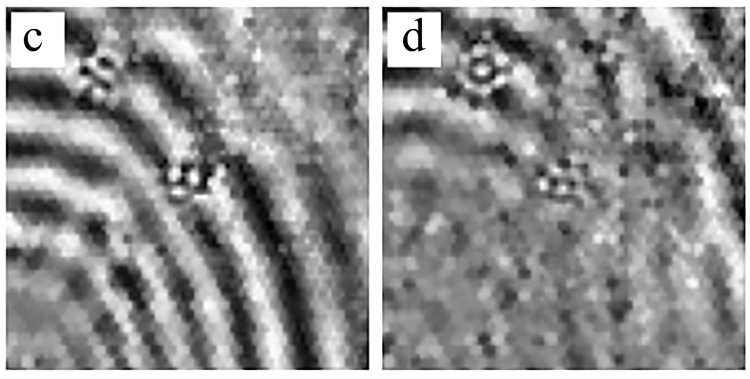
Experimental results at the frequency 45 kHz for D2 with two debondings at the locations of (100,100) and (50,164): (**a**) wave field at 20 μs; (**b**) wave field at 160 μs; (**c**) wave field at 300 μs; (**d**) wave field at 440 μs.

The results of multi-damage zones detection at the excitation frequency of 45 kHz are presented in Figure 10. At 20 μs, the waves of quasi-$A_{0}$ mode were generated from the PZT actuator and the plate as covered by quasi-$S_{0}$ mode that exhibited a higher velocity and weak out-of-plane component in Figure 10a. At 160 μs, the quasi-$S_{0}$ mode waves with weak out-of-plane component interacted with debondings. Subsequently, the mode conversions occurred and reflected waves appeared, which were caused by debonding edges in Figure 10b. The reflected waves exhibited much stronger out-of-plane vibration components than the quasi-$S_{0}$ incident mode waves. The quasi-$A_{0}$ mode waves arrived at the debondings at 300 μs in Figure 10c. The incident quasi-$A_{0}$ mode exhibited strong wave interactions between the damage and the reflected waves which were conversed by quasi-$S_{0}$ mode. Furthermore, the interactions between the reflected waves and the indicated waves formed new patterns around the damage zones, which were manifestly different from the SWIs in the perfect zones. The clearly reflected waves, which were caused by damages, gradually submerged at 440 μs, as seen in Figure 10d. The wave fields of the scan area measured by SLV displayed the multi-damage zones in the course of the process of wave propagation.

**Figure 11:**
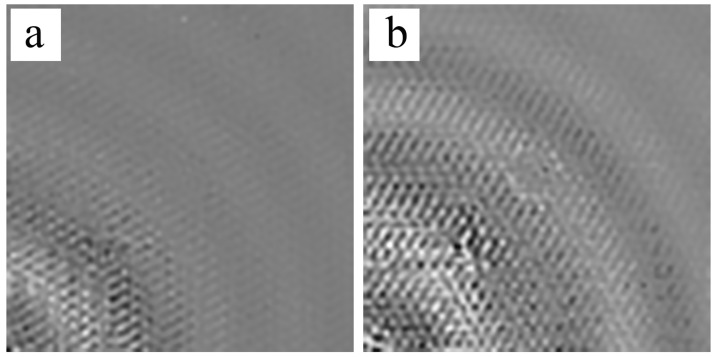

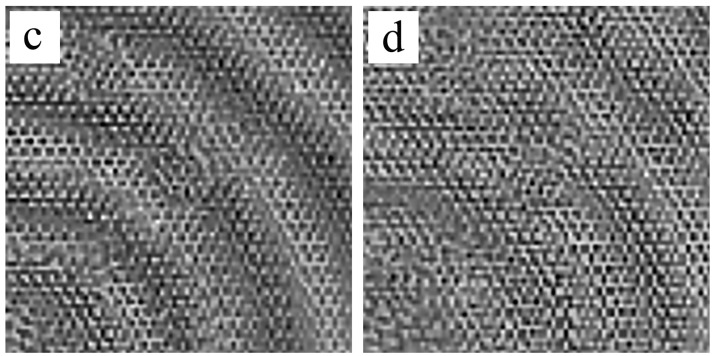
Experimental results at the frequency 80 kHz for D2 with two debondings at the locations of (100,100) and (50,164): (**a**) wave field at 12 μs; (**b**) wave field at 52 μs; (**c**) wave field at 92 μs; (**d**) wave field at 132 μs.

Figure 11 shows the process of GWs propagating in the scan area of the D2 measured by SLV. Because of the mode-tuning affection of PZT, the energy distribution in different mods changed according to varying frequencies. The quasi-$A_{0}$ mode was most pronounced at a low-frequency band, while the quasi-$S_{0}$ mode was more dominant at higher frequencies [29]. Although the quasi-*S*_0_ mode was the dominant mode at high frequencies, its out-of-plane vibration was minimal. Therefore, even the energy proportion of the quasi-$S_{0}$ mode in the GWs increased with the frequency, while the out-of-plane vibration component obtained by SLV belonged to quasi-$A_{0}$ mode. In Figure 11a, the wave field consisted of the quasi-$A_{0}$ mode with strong incident wave fronts near the PZT and the quasi-$S_{0}$ mode with weak wave fronts covering the rest of the figure at 12 μs. Under the excitation frequency of 80 kHz, the wave field of the scan area showed several circular wave fronts at the macroscopic level. Each wave-front comprised many SWIs that were induced by the cellular microstructures of the HSSs. At 52 μs, the incident waves arrived at the debonding A and the wave fronts were interrupted by the debonding, as seen in Figure 11b. No SWIs occurred in the damage zone of debonding A. The reflected waves superimposed the incident waves, showing a new wave field, which was demonstrable via a comparison of the different patterns to the perfect areas. The waves exhibited no complex SWIs in the debonding zone. At 92 μs and 132 μs, the wave field in the debondings was not outstanding compared with the undamaged areas because of the reflected waves and scattering waves shown in Figure 11c,d.

**Figure 12:**
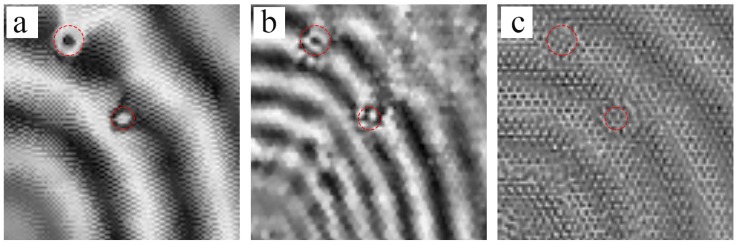
Results of debonding detection experiments at different excitation frequencies: (**a**) 15 kHz at 400 μs; (**b**) 45 kHz at 300 μs; (**c**) 80 kHz at 92 μs.

**Figure 13:**
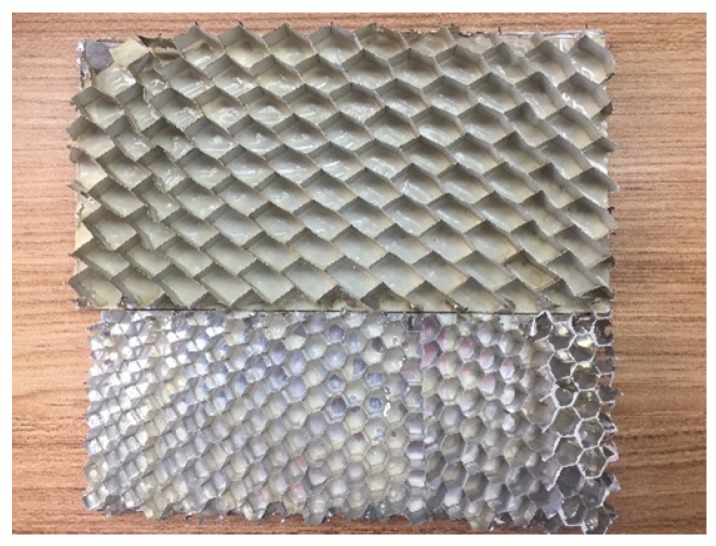
The deformed honeycomb cores during the fabrication.

In Figure 12, the red circle indicates the real location of debonding A and B. The undamaged zones in honeycomb plates show SWIs consisting of regular patterns at 45 kHz and 80 kHz, which correspond to the numerical simulations in Figure 5 and Figure 6, respectively. Moreover, the wave fields of the damaged zones showed new patterns which were caused by strong reflected waves from the debonding edges, representing damages. The new patterns were different from the regular patterns in the undamaged zones. Therefore, it was unnecessary to refer to the reference samples. The identified damage zones mostly cover the real debonding zones in red circles under all three excitation frequencies. But the damaged zones are insignificant because the regions of debondings show less impact at 80 kHz. The second-order mode shape complicated the patterns, disturbing the reflected waves which represented the damage regions. Therefore, the excitation frequency should be below the second-order natural frequency of the hexagonal surfaces. Furthermore, the SWIs patterns could identify not only debondings but also core deformations or non-uniformed shapes in the inner honeycombs, as shown in Figure 13.

In comparison with the actual dimensions, the identified damage areas were larger than the real debonding zones. Two factors account for this. Firstly, the instrumental errors may have affected the results of the experiment. For example, the spatial resolution of SLV was approximately 2.5 mm. In addition, the limitation of the spatial resolution caused the overlapping curves in the wave fronts in Figure 8a and Figure 9. Secondly, the edges of the completely debonded area caused the adjacent hexagons to partially debond; as a result, the real area of debondings was larger than the prescribed dimensions. Although the errors were unavoidable, the positions and sizes of damages were identified.

## 5. Conclusions

The predominant features of interactions between GWs and HSSs is investigated in the present study. To study the relationship between the excitation frequency and the geometry parameters of HSSs, the finite element method was used to simulate the wave propagation. A method based on the phenomenon of SWIs was developed to detect skin-core debondings in the HSSs. Scanning laser vibrometry experiments were performed to verify the simulation results of SWIs. Multiple debondings were identified experimentally.

The SWIs that occur synchronously with cellular microstructures of HSSs were the unique feature to distinguish the perfectly bonded honeycomb zones from the debonding zones. To better utilize the SWIs for debonding estimation, it is crucial to select an appropriate excitation frequency. An optimal excitation frequency was determined to be slightly higher than the first-order natural frequency of the hexagon (45 kHz). The results of the patterns in wave fields which were measured by SLV agreed with the simulation results. On the basis of the phenomenon of SWIs, the sizes and locations of debondings were assessed and the results were compared with the actual parameters to prove the reliability of the technique. Because of the limitations in resolution of the SLV, several features and mode shapes of the hexagonal surface were indistinct. In further studies, more powerful equipment and digital image processing procedures could be employed to enhance the detected images.

## Figures and Tables

**Table 1:** Geometry parameters of the finite element model (unit: mm).

Skin Panels			Honeycomb Core			PZT Actuator	
Length	Width	Thickness	Cell Size	Wall Thickness	Height	Diameter	Thickness
290	300	0.3	5	0.03	19.7	10	0.3

**Table 2:** The first ten order natural frequencies of the hexagon surface of the honeycomb.

Order	Frequency (kHz)	Order	Frequency (kHz)
1	37.59	6	186.75
2	77.06	7	210.47
3	124.15	8	238.09
4	141.18	9	289.80
5	171.13	10	297.32

**Table 3:** Summary of debondings for HSSs.

Sample	Debonding Location (mm)	Debonding Size (mm)
Debonding A	(100,100)	20
Debonding B	(50,164)	26
